# Integrated transcriptomics and metabolomics analyses of the effects of bagging treatment on carotenoid biosynthesis and regulation of *Areca catechu* L.

**DOI:** 10.3389/fpls.2024.1364945

**Published:** 2024-04-02

**Authors:** Xin Zheng, Liyun Huang, Benyi Fan, Chunlin Peng, Amjad Iqbal, Yujie Zhang, Hongman Chen, Jianqiu Ye, Yaodong Yang

**Affiliations:** ^1^ Coconut Research Institute, Chinese Academy of Tropical Agricultural Sciences, Wenchang, China; ^2^ National Nanfan Research Institute, Chinese Academy of Agricultural Sciences, Sanya, China; ^3^ Department of Food Science & Technology, Abdul Wali Khan University Mardan, Mardan, Pakistan; ^4^ Planting Research Section, Hainan Agriculture School, Haikou, China

**Keywords:** *Areca catechu* L., bagging, carotenoid, metabolomics, transcriptomics

## Abstract

**Introduction:**

Fresh Aareca nut fruit for fresh fruit chewing commonly found in green or dark green hues. Despite its economic significance, there is currently insufficient research on the study of color and luster of areca. And the areca nut fruits after bagging showed obvious color change from green to tender yellow. In the study, we tried to explain this interesting variation in exocarp color.

**Methods:**

Fruits were bagged (with a double-layered black interior and yellow exterior) 45 days after pollination and subsequently harvested 120 days after pollination. In this study, we examined the the chlorophyll and carotenoid content of pericarp exocarp, integrated transcriptomics and metabolomics to study the effects of bagging on the carotenoid pathway at the molecular level.

**Results:**

It was found that the chlorophyll and carotenoid content of bagged areca nut (YP) exocarp was significantly reduced. A total of 21 differentially expressed metabolites (DEMs) and 1784 differentially expressed genes (DEGs) were screened by transcriptomics and metabolomics. Three key genes in the carotenoid biosynthesis pathway as candidate genes for qPCR validation by co-analysis, which suggested their role in the regulation of pathways related to *crtB, crtZ* and *CYP707A*.

**Discussion:**

We described that light intensity may appear as a main factor influencing the noted shift from green to yellow and the ensuing reduction in carotenoid content after bagging.

## 1 Introduction

*Areca catechu L*., a perennial evergreen tree, stands out as one of the economically viable species within the palm family. The fresh areca nut is green, ovoid, generally measuring about 3 to 4 cm in width and 4 to 5 cm in length. As an important economic tree species in tropical and subtropical regions, A. catechu is native to Malaysia and is mainly distributed in South Asia, Southeast Asia and the Pacific Islands (Heatubun et al., 2012). In China, the main producing areas of areca nut are Hainan and Taiwan, and there are also small amounts of cultivation in Guangdong, Guangxi, Fujian, and some parts of Yunnan Province (Zhao et al., 2006). In recent years, the areca nut industry has emerged as one of the most important pillars of tropical agriculture in Hainan.

A. catechu is highly famous among the population, with individuals frequently chewing it in both fresh fruit and dried fruit forms. It is worth mentioning that the tradition of fresh areca nut chewing has been prevalent in Southeast Asian countries, Hainan, Taiwan and other places for a long time (Huang et al., 2014). Like other fruits, for fresh areca nut, the color of the fruit, which significantly influences the commodity’s value, is one of the most crucial attributes in the external quality of plant fruits. Peels that are bright and free of blemishes are generally acceptable by the consumers, resulting in an increased commodity value. According to relevant reports, the color of most fruits is mainly determined by the chlorophyll, carotenoid and anthocyanin. Generally, higher chlorophyll content results in green peel, while high carotenoid content leads to yellow, orange or red peel. Likewise, high anthocyanin content yields bluish-purple or black color (Wang, 2022). At present, there is no existing report on the color of areca nut.

To inspect the patterns of gene expression and metabolite changes in the development of areca nut rind color, and studying the practical simplicity of the process, we have chosen the bagging method from among various techniques. Bagging is an important technical measure for the production of high-quality fruit, and different bagging treatments affect different fruit phenotypic traits. In general, the effect of bagging on the exocarp color is more intuitive. The use of a common bagging type, such as an outside yellow-inside black paper bag, has been studied and shown to affect the color of avocado, peach, pear, pomelo and citrus (Li Q. et al., 2017; Cheng et al., 2017; Li et al., 2018; Wu et al., 2020; Liu et al., 2022). In addition, we integrated transcriptomics and metabolomics analyses in an attempt to explore areca nut pericarp coloration more deeply at the molecular level. This technology has been used extensively and become a relatively mature and cutting-edge technology, playing a key role in the aspect of plant fruit color research in recent years. Numerous studies have applied this technology to elucidate color studies in fruits such as papaya, longan, figs, etc. These efforts have laid a foundation for the improvement of fruit appearance and quality (Wang et al., 2017; Shen et al., 2019; Yi et al., 2021). Currently, research on areca nut fruit histology involves exploring fruit developmental rules, studying fruit nutrients (flavonoids, vitamin B, etc.), while there is still in the relative blank stage of exploring areca nut exocarp color (Ya et al., 2020; Yu et al., 2023; Zhou et al., 2023).

**Figure 1:**
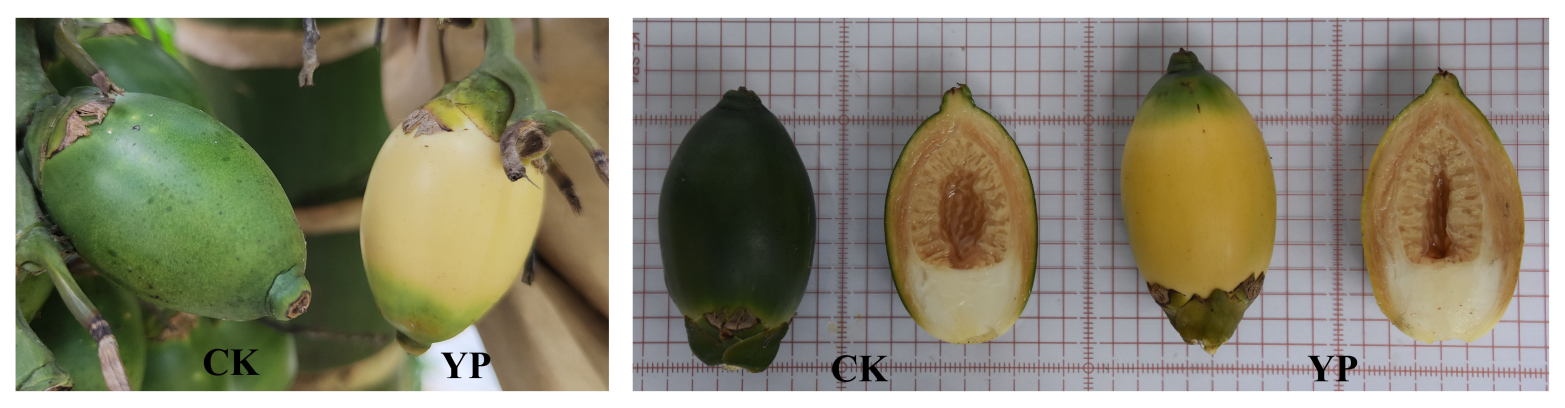
CK (untreated control) vs YP (bagged areca nut).

Based on previous studies and considering the growth habits of areca fruit, we attempted to use a paper bag with yellow on the outside and black on the inside (providing a shading rate of 99.88%) to manage areca fruit after 45 days of pollination. Subsequently, harvesting was performed during the commercial harvest period (120 days after pollination). The goal was to offer a more comprehensive explanation for the notable and intriguing transformation by integrating phenotypic measurements and pigment content analysis through advanced plant transcriptomics and metabolomics techniques. This study specifically focuses on changes in carotenoids in the exocarp due to the yellow color of YP (**Figure 1**). The YP was also compared with the untreated control (CK) to analyze the effects of bagging treatment on its biosynthesis and regulation.

## 2 Materials and methods

### 2.1 Plant materials and bags

Healthy “Reyan No. 1” were collected from the areca germplasm resources nursery of the Coconut Research Institute, Chinese Academy of Tropical Agricultural Sciences (Wenchang, Hainan Province; longitude 110gitude,a E, latitude 19titudee, N) as experimental material. Three plants with relatively uniform growth were selected from different directions of the site. For each selected plant, 20-30 fruits on the bunches were bagged, while the untreated portion was used as CK. Harvesting was conducted 120 days after pollination for subsequent experiments.

The double-layer inner black and outer yellow paper bags selected for the test measured 31 × 23cm and were purchased from Green Farmers Fruit Bags.

### 2.2 Bagging method and operation

About 45 days after pollination of areca nut female flowers, spray insecticide to kill insects, the insecticide used is thiamethoxam and chlorpyrifos mixed application, thiamethoxam concentration of 0.1%, chlorpyrifos concentration of 0.15%. Then hang the pest avoidance agent bag, the pest avoidance agent is p-dichlorobenzene (content 99%), hanging the pest avoidance agent into the bag tied to the near set of fruit stalks, bagging, the pest avoidance agent bag together in the bag. Cut off the male flowering branches at the front end of the spike before bagging, and the fruits need to be harvested about 120 days after pollination when the bags can be removed.

### 2.3 Fruit phenotype and chromaticity measurements

The longitudinal diameter, transverse diameter, and weight of the freshly harvested areca nut, as well a color difference of exocarp, were measured and presented as average values.

The chromaticity color parameters of the exocarp include L^*^, a^*^ and b^*^. The fruit surface color composite index (CCI) was utilized to indicate color saturation, and was calculated by the formula CCI =(1000 × a*)/(L* × b*). L* denotes brightness, a^*^ value represents the red-green color difference of the exocarp, and b^*^ value represents the yellow-blue color difference of the exocarp (Wang et al., 2023).

### 2.4 Fruit exocarp pigment extraction and content measurement

The extraction method of chlorophyll and carotenoid content of areca nut exocarp was done according to the method described by previous research (Li S. et al.,2017). Three portions of fresh areca nut exocarp were weighed from both the YP and CK (mixed sample of fruit exocarp, 0.5 g each). Initially, calcium carbonate and quartz sand were used for grinding. Then the powder was further ground with liquid nitrogen and extracted using 80% acetone.

### 2.5 Extraction and analysis of metabolites

Following the freeze-drying of areca nut fruit exocarp samples in a vacuum freeze dryer (Scientz-100F), the lyophilized exocarp samples were ground (30 Hz,1 min) to powder using a ball mill (MM 400, Retsch). Subsequently, 50 mg of each sample was weighed and extracted with 0.5 mL of a hexane/acetone/ethanol mixture (1:1:1, v/v/v) containing 0.01% BHT (g/mL). After vortexing (room temperature) for 20 min, the mixture was centrifuged (4 °C, 12000 rpm) for 5 min. The supernatant was collected and the extraction was repeated once, mixing the resulting supernatant. The collected supernatants were concentrated, re-dissolved with methanol/methyl tert-butyl ether mixture (1:1, v/v, 100 μL), passed through filter membrane (0.22 μm), and consequently stored in a brown sample bottle for LC-MS/MS analysis. The data acquisition instrumentation system primarily consisted of Ultra Performance Liquid Chromatography (UPLC) (ExionLC™AD, https://sciex.com.cn) and Tandem Mass Spectrometry (MS/MS) (QTRAP^®^6500+, https://sciex.com.cn/) (Inbaraj et al., 2008; Zhao et al., 2022).

LC was performed according to the following conditions: column: YMC C30 (3 μm, 100 mm × 2.0 mm i.d.); mobile phase: A phase = methanol/acetonitrile (1:3, v/v) with 0.01% BHT and 0.1% formic acid; B phase = methyl tert-butyl ether with 0.01% BHT; gradient elution program: 100:0 (V/V) for 0 min A/B, 100:0 (V/V) for 3 min, 30:70 (V/V) for 5 min, 5:95 (V/V) for 9 min, 100:0 (V/V) for 10 min, and 100:0 (V/V) for 11 min. The flow rate, column temperature, and injection volume were 0.8 mL/min, 28 °C, and 2 μL, respectively.

The mass spectrometry conditions consisted primarily of an atmospheric pressure chemical ionization source (APCI, 350 °C) and curtain gas (CUR, 25 psi). In the Q-Trap 6500+, per ion pair was scanned and detected based on optimized de-clustering potential (DP) and collision energy (CE) (Mase et al., 2022).

Carotenoid standards at various concentrations were prepared, and the mass spectral peak intensities of the quantitative signals of each concentration were obtained for each concentration. Standard curves were plotted using concentration as the horizontal coordinate and area as the vertical coordinate. The content of the substance in the samples were calculated using the linear equation of the standard curve. The difference in the amount of each substance in different groups was shown by drawing bar charts. DEMs with criteria of |log2 (FoldChange)|≥1 and p-value<0.05.

### 2.6 Transcriptome sequencing analysis

The total RNA of the areca exocarp was used to obtain the required mRNA for library construction. Enrichment of mRNA containing polyA tails was performed with Oligo (dT) magnetic beads. To construct the library (NEBNext^®^ Ultra™ RNA Library Preparation Kit for Illumina^®^), the first strand of cDNA was synthesized in the M-MuLV reverse transcriptase system using fragmented mRNA as a template and random oligonucleotides as primers. Subsequently, the RNA strand was degraded with RNaseH and the second strand of cDNA was synthesized with dNTPs in the DNA polymerase I system. Thereafter, the purified double-stranded cDNA was subjected to end repair, A-tailing and ligation to sequencing junctions. The cDNA (250-300 bp) were screened with AMPure XP beads and PCR amplified, and the products were purified again using AMPure XP beads, ultimately resulting in the libraries.

Initial quantification was performed using a Qubit 2.0 fluorometer, and the library was diluted to 1.5 ng/μl. Later, the insert size of the library was detected using an Agilent 2100 Bioanalyzer. After confirming size compliance, the effective concentration of the library was accurately quantified utilizing qRT-PCR to ensure that the quality of the library met the required standards for an effective concentration higher than 2 nM. Sequencing was performed on an illumina NovaSeq 6000 (illumina, USA).

Quantitative analysis was performed using the featureCounts tool in the subread software, based on information about the position of the gene ratio on the reference genome, thus counting the number of reads covered by each gene from the start to the end of the range, and filtering out reads with a ratio quality value of less than 10. Differential analysis of gene expression was performed using DESeq 2 software. GO functional enrichment analysis and KEGG (Kyoto Encyclopedia of Genes and Genomes) pathway enrichment analysis of differential gene sets using clusterProfiler software. DEGs with criteria of |log2 (FoldChange)|≥1 and padj<0.05.

### 2.7 Integrated transcriptomics and metabolomics analyses

Correlation analysis was performed by comprehensive screening of exocarp carotenoid-related DEMs and DEGs using quantitative values of genes and metabolites in all samples. The correlation method was to calculate Pearson correlation coefficients of genes and metabolites using cor function in R. Correlation coefficients greater than 0.80 with a pvalue of less than 0.05 correlation result was selected for further discussion and analysis. According to the results of KEGG enrichment analysis, the annotated DEGs and DEMs were simultaneously mapped to the carotenoid-related KEGG pathway.

### 2.8 RT-qPCR

The total RNA from the areca exocarp was extracted by a RNAprep Pure polysaccharide polyphenol plant total RNA extraction kit (DP441, TianGen, China). Total RNA concentration, quality and integrity were assessed using NanfoDrop 2000 (Thermo, USA) and gel electrophoresis. High-quality total RNA as a template for cDNA synthesis using the PastKing One-step Synthesis of Premixed Reagents by Removing the First Chain of Genomic cDNA (KR118-02, TianGen, China). After the initial screening of DEMs and DEGs, we combined correlation coefficients (>0.8) and KEGG annotations, focusing on the upstream and downstream of the major DEMs, and screened the key genes in the carotenoid biosynthesis pathway as candidate genes for qPCR validation. We used the housekeeping gene (Acactin) as an internal reference gene and the relative expression of the genes was calculated using the relative quantification method (2^-ΔΔCT^). The primers involved in qPCR are shown in **Supplementary Table 3**.

### 2.9 Statistical analysis

In this study, SPSS 26.0 (IBM Corporation, USA) was used for statistical analysis of data, and Excel 2016 (Microsoft, USA) was used for data collation. GraphPad Prism 9.5 (GraphPad Software, USA), the online bioinformatics platform (http://www.bioinformatics.com.cn) and Adobe Illustrator CS6 (Adobe, USA) software were used for graphical representation.

## 3 Results

### 3.1 Fruit basic physical parameters, exocarp color and pigment content

**Figure 2:**
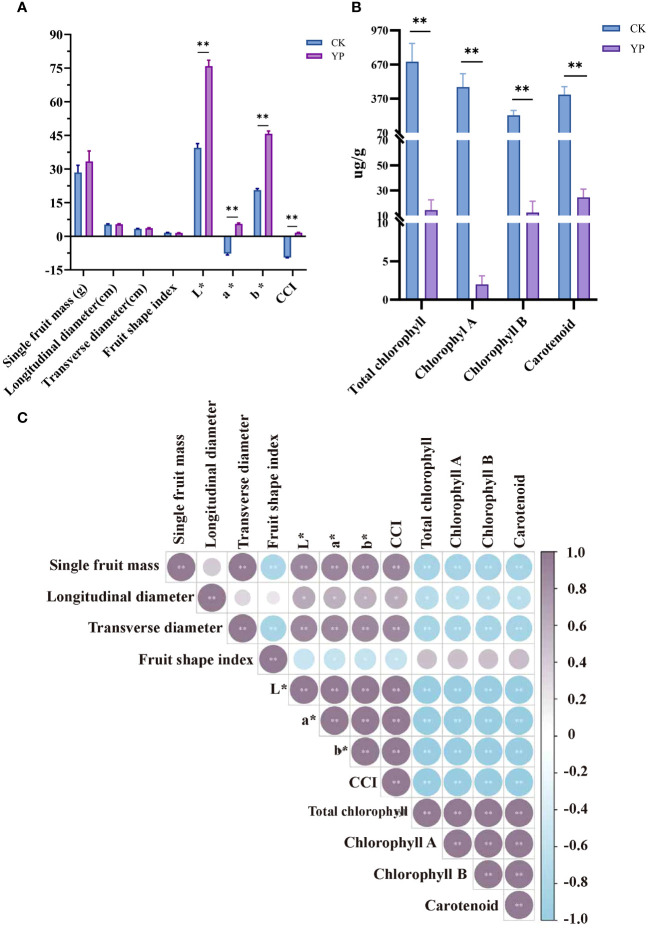
**(A)** Effect of changes in basic phenotypic indexes and color parameters L*, a*, b*, and CCI of bagged areca nut (YP). L* denotes brightness, a*value represents the red-green color difference of the exocarp, and b* value represents the yellow-blue color difference of the exocarp. Color composite index (CCI) was utilized to indicate color saturation. **(B)** Areca exocarp pigment content. **(C)** Pearson correlation coefficients of areca nut measurements. Fruit shape index is the ratio of longitudinal diameter to transverse diameter of the fruit. All the ** indicates highly significant correlation above (p<0.01).

Measurements of areca nut fruit weight, transverse diameter and longitudinal diameter and coloration are shown in the **Figure 2A**. And measurements of areca nut exocarp pigment content are shown in the **Figure 2B**. The single fruit weight, longitudinal diameter, transverse diameter and fruit shape index of the bagged treatments (YP) were 33.38 g, 5.35 cm, 3.60 cm and 1.49, respectively, while several indices of CK were 28.43 g, 5.22 cm, 3.33 cm and 1.57, respectively, and the differences were not significant. However, the results of indicators related to chroma are completely different, and all indicators (L*, a*, b* and CCI) have reached highly significant levels compared with CK (p < 0.01).

We compared the contents of CK and YP pigments. Based on the results shown regarding the pigment content of the exocarp both chlorophyll and carotenoid contents exhibited a sharp decrease after bagging treatment, reaching to highly significant levels (p < 0.01). It is worth mentioning that most pronounced decrease was observed in chlorophyll A content, dropping from an average value of 471.52 μg/g to 1.99 μg/g. The change between YP and CK even reached 237 times. The total chlorophyll content showed a significant reduction (695.43 μg/g vs. 14.41 μg/g), chlorophyll A (471.52 μg/g vs. 1.99 μg/g), chlorophyll B (224.06 μg/g vs. 12.48 μg/g) and carotenoid (405.62 μg/g vs. 24.50μg/g) changing by 48, 236, 18 and 16 times, respectively (**Figure 2B**).

In terms of correlation between indicators (**Figure 2C**), the correlation of single fruit mass with transverse diameter and fruit shape index was highly significant (p < 0.01). Additionally, several indicators, including L*, a*, b* and CCI, all parameters related to chromaticity, as well as the total chlorophyll, chlorophyll A, chlorophyll B, and carotenoids pigments, exhibited highly significant correlation coefficients with each other (p < 0.01).

### 3.2 Metabolomics analysis of areca exocarp

**Figure 3:**
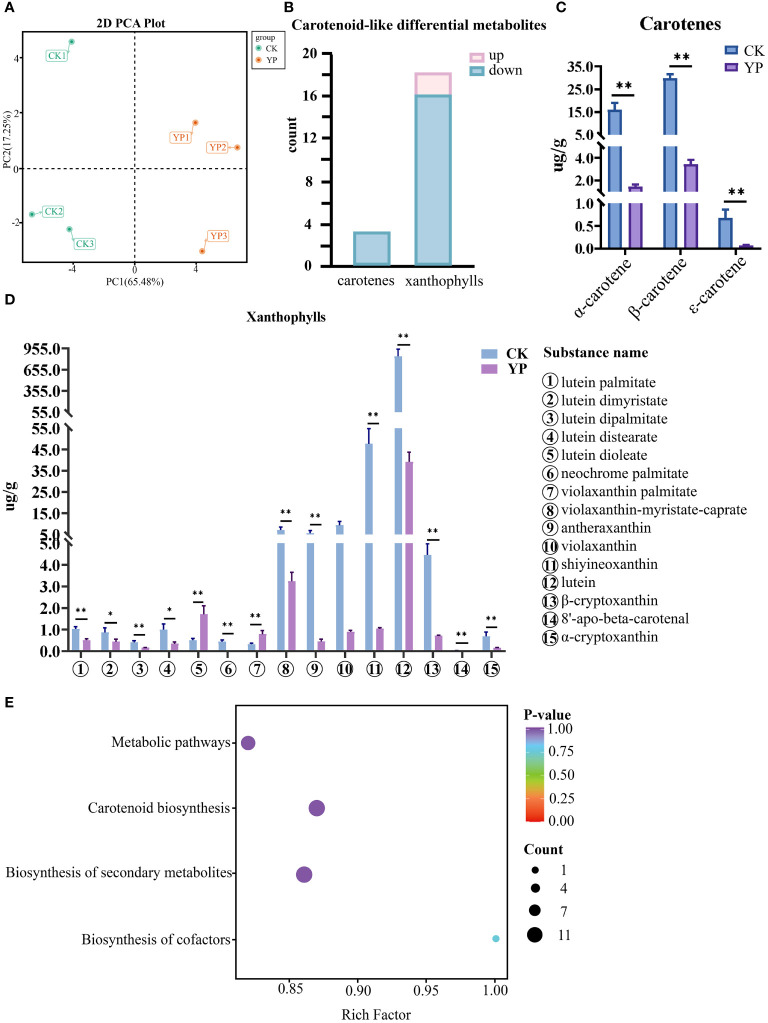
**(A)** Principal component analysis (PCA) of untreated (CK) and bagged (YP) samples. **(B)** Differential metabolite statistics of carotenoids in areca nut exocarp. **(C)** Changes in carotenes class (top3) content of areca nut exocarp after bagging treatment. **(D)** Changes in xanthophylls class (top15) content of areca nut exocarp after bagging treatment. The * means p<0.05 for significant correlation and ** means p<0.01 for highly significant correlation. **(E)** Differential metabolite KEGG enrichment map. The horizontal axis indicates the ratio of the number of differential genes under the pathway to the total number of differential genes, and the vertical axis indicates the descriptive information of the pathway enriched to.

In this study, we examined 68 carotenoids in areca nut exocarp like α-carotene, β-carotene, and lutein. Furthermore, the principal component analysis (PCA) of two groups, the untreated exocarp and the exocarp of bagged samples are denoted as CK and YP, respectively. The variances of the first and second principal components were 65.48% and 17.25%, respectively. This clearly revealed a significant separation of areca nut exocarp metabolites before and after bagging treatment, with a certain consistency perceived between the three replicates (**Figure 3A**). Hence, the data involved in this study can be used for further analytical exploration.

The results of the screening showed that there were 21 carotenoid-like DEMs in the epidermis of areca nut fruits between the CK and YP, the numbers of up-regulated and down-regulated metabolites were 2 and 19, respectively (**Figure 3B**).

Interestingly, the results show that the vast majority of DEMs were down-regulated after bagging, with all three screened carotenoids (α-carotene, β-carotene, ϵ-carotene) being highly significantly down-regulated (P<0.01) compared to CK. The two up-regulated species (lutein dioleate, violaxanthin palmitate), both belonging to the lutein class, also exhibited highly significantly different compared with CK. Except for lutein dimyristate and lutein distearate, which showed significant differences (P<0.05) from the control. The differences between the YP and CK for the rest of the screened metabolites of lutein class reached highly significant levels (P<0.01) (**Figure 3C**).

Among the screened DEMs, it is noteworthy that lutein had the highest metabolic expression in both CK and YP exocarp when harvested 120 days after pollination. However, concerning the mean value, the expression after bagging was narrowed to 21.5-fold that of CK, with the mean expression value decreasing from 844.62 to 39.20. Although the expression of neoxanthin was second only to that of lutein, the numerical difference between the two substances was still very intuitive. The decrease of neoxanthin after bagging was the most prominent among all DEMs, and the difference between the mean values of the CK group and those of the bagged YP group could reach 45.8-fold, with the mean expression value decreasing from 47.71 to 1.04. In addition, the metabolites such as β-carotene, α-carotene, and violaxanthin were relatively highly expressed in the CK group, with mean expression values of 29.72, 16.07, 9.36, respectively. However, after bagging, the reductions in these examples reached 8.7-fold, 11.1-fold, and 10.4-fold, respectively. Among the rest of the screened DEMs, antheraxanthin decreased by more than 10-fold (**Figure 3D**). Annotation results for the DEMs were categorized by pathway type in KEGG, and KEGG pathway enrichment analysis showed that four pathways (metabolic pathways, biosynthesis of secondary metabolite, carotenoid biosynthesis and biosynthesis of cofactors) were involved in two comparison groups(CK and YP) (**Figure 3E**).

### 3.3 Transcriptomics analysis of areca exocarp

**Figure 4:**
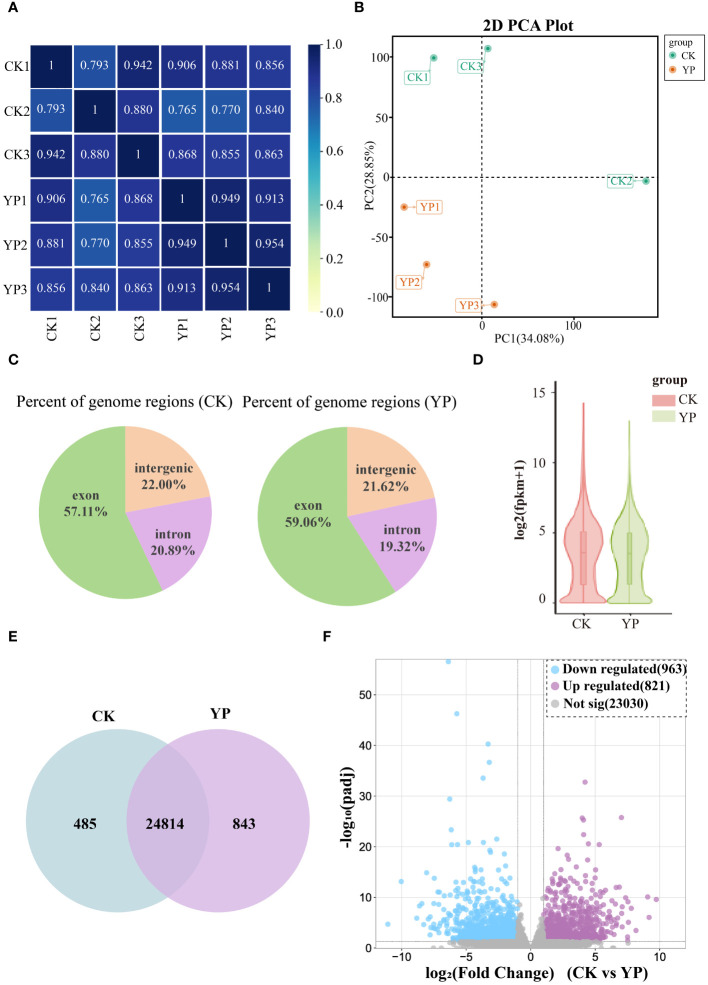
**(A)** Pearson correlation between untreated (CK) and bagged (YP) samples. **(B)** Principal component analysis (PCA) of the transcriptome of CK and YP samples. **(C)** Percent of genome regions of different examples. **(D)** Gene expression level violin map. The width of the violin plot indicates the horizontal expression of genes, and each region corresponds from bottom to top to the five statistics of minimum, lower quartile, median, upper quartile and maximum values. **(E)** Venn diagram of genes in CK and YP. **(F)** Volcano plot of differentially expressed genes (DEGs) between CK and YP. Blue dots, purple and gray dots indicate down-regulation, up-regulation and non-significant, respectively.

In this study, we aimed to utilize the transcriptomics to comprehend the genetic mechanisms underlying the differences in metabolite content of areca nut exocarp after bagging. As observed from the correlation heat map, which was analyzed for all samples based on transcriptome comparisons, the high data for each sample suggests that transcriptomics data between two groups (CK and YP) were well accurate and reliable (**Figure 4A**). The PCA showed significant sample separation between the YP group and the CK group, with greater variation within the CK group than the YP group (**Figure 4B**). This is consistent with the metabolomics data, suggesting that the accumulation of DEMs after bagging is regulated by the DEGs. The percentage of genomic regions occupied by different instances of the samples in this study is reasonable (**Figure 4C**). And the overall profile of gene expression levels of the samples in this study is presented as a violin plot (**Figure 4D**), with similar interquartile ranges and medians between the CK and YP groups, with the 95% confidence intervals for the latter being relatively more clustered.

Comparing the gene expression revealed that 24814 genes were co-expressed between the CK groups and YP groups, with the number of genes expressed in each of the two groups was 485 and 843, respectively (**Figure 4E**). In addition, volcano plots were generated to visualize the DEGs between two groups. A total of 1784 DEGs were identified from the two groups of samples, 821 genes were up-regulated and 923 down-regulated (**Figure 4F**).

Out of the 1784 DEGs, a total of 713 (40%) were annotated to different transcription factor families (Pkinase, p450 and Pkinase_Tyr etc.). 

**Figure 5:**
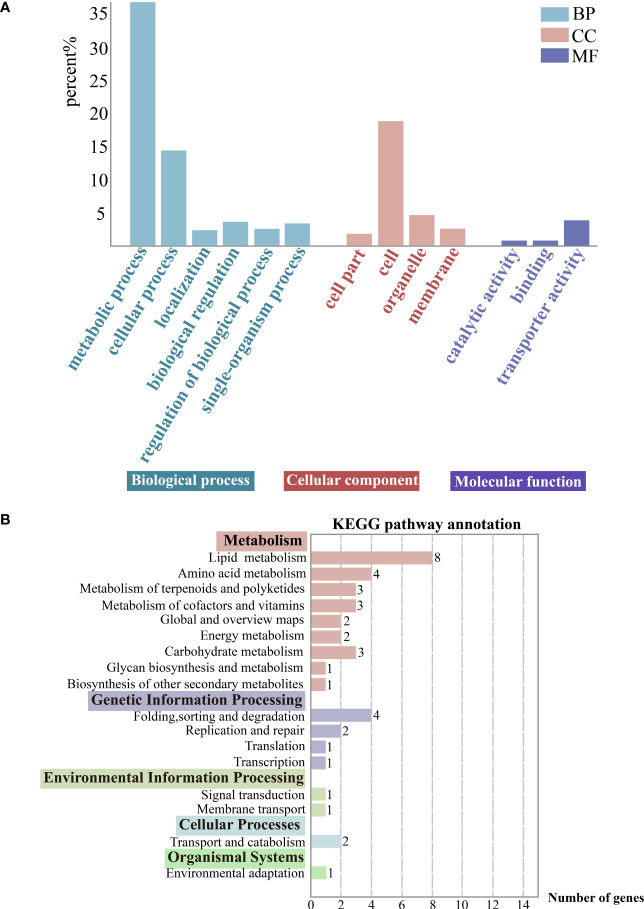
**(A)** Gene function classification (GO). Horizontal coordinates indicate go-term categories and vertical coordinates indicate the percentage of each category of differentially expressed genes (DEGs) to the total DEGs. **(B)** KEGG enrichment analysis of the DEGs. The color-bordered black font next to the vertical coordinates indicates the KEGG pathway primary classification, and the rest are the corresponding secondary classifcations. Horizontal coordinates indicate the DEGs counts enriched in this pathway. And the image showing distribution of KEGG primary and secondary pathways.

The 1784 DEGs obtained were further enriched and analyzed using the GO (Gene Ontology) database, and the DEGs were annotated on GO terms in three categories, namely Biological process, Cellular component and Molecular function. We chose the categories with a high percentage of GO secondary entries, in terms of Biological process, the main secondary classifications mainly include metabolic process (GO: 0008152), cellular process (GO: 0009987), localization (GO: 0051179), single-organism process (GO:0044699), and regulation of biological process (GO:0050789) in terms of Biological Process. In terms of Cellular components, they mainly include cell part (GO: 0044464), cell (GO: 0005623), organelle part (GO: 0044422), and membrane (GO: 0016020). In terms of Molecular function the main categories are catalytic activity (GO:0003824), binding (GO:0005488), and transporter activity (GO:0005215). Particularly, metabolic process genes are active (**Figure 5A**).

DEGs were categorized using the KEGG database, and they were annotated on primary (5), secondary (17), and tertiary pathways (40). And the DEGs were mostly enriched in metabolic pathways. Specifically, there was a focus on the carotenoid biosynthesis pathway (ko00906), which belongs to the metabolism of terpenoids and polyketides and is relevant to exocarp color (**Figure 5B**).

### 3.4 Integrated transcriptomics and metabolomics analyses

Totally, 11 structural genes were annotated for five enzymes in the carotenoid biosynthesis pathway. Subsequently, we conducted further analysis by combining the transcriptomics and metabolomics, correlation analysis was performed using quantitative values of genes and metabolites in all samples. The Pearson correlation coefficients for the genes and metabolites were calculated using the cor function in R, and from these, results greater than 0.80 and with a p-value less than 0.05 were selected. Considering the correlation coefficient, the value of |log2 (FoldChange)|, and the major metabolites, three candidate genes, namely Acat_3g005410, Acat_3g016820 and Acat_9g010750 from the DEGs were selected for further validation.

**Figure 6:**
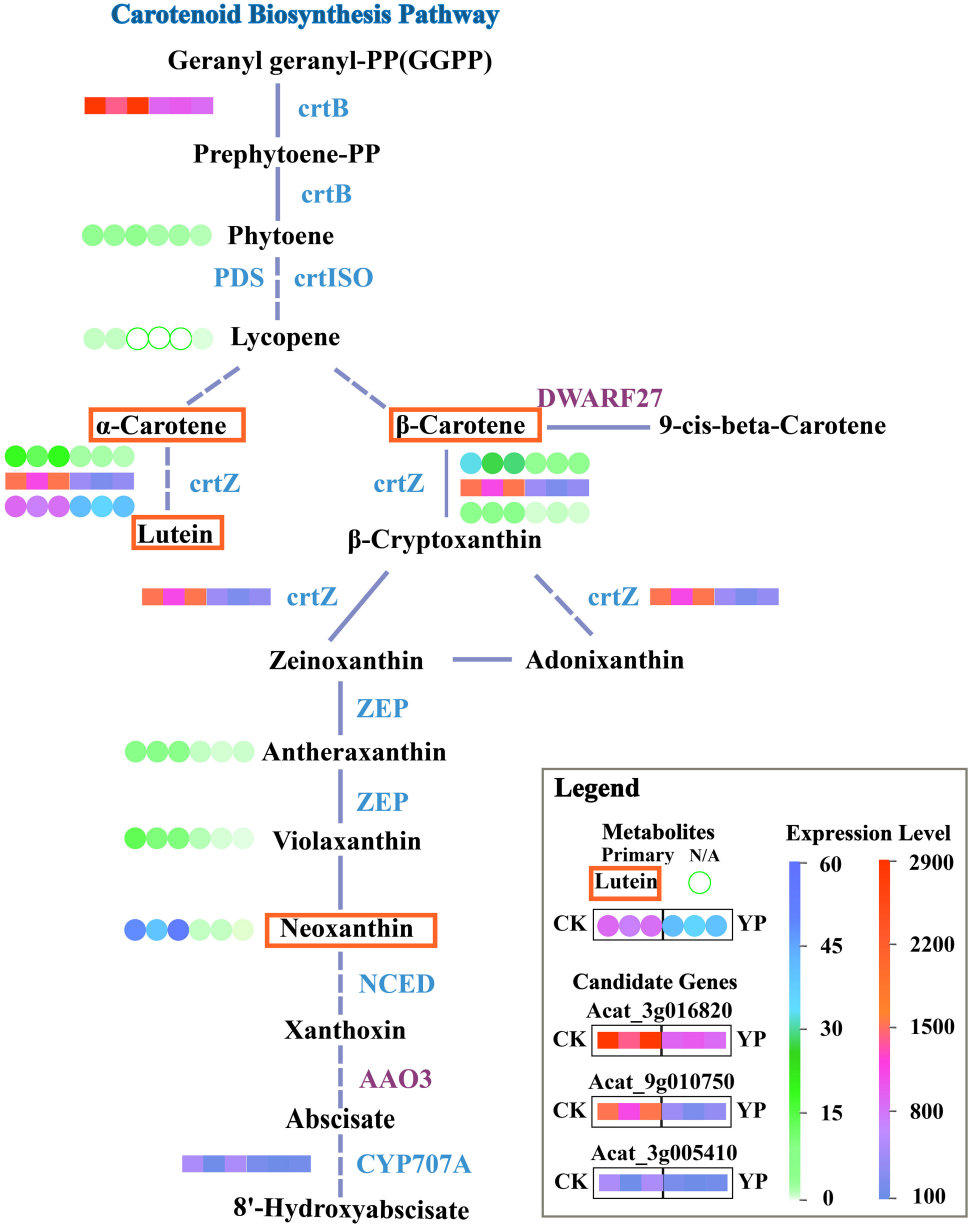
Simplified KEGG pathway map for carotenoid biosynthesis. Dashed lines indicate that direct pathways of metabolites have been omitted and solid segments indicate that steps have not been omitted. Blue lettering nest to the line segments indicates down-regulation of genes, purple indicates up-regulation. Candidate genes expression profiles in the pathway map are transcriptome results and metabolite profiles are metabolome results. Expression of candidate genes after bagging (YP) versus untreated (CK) is indicated by colored squares. Metabolites are indicated by colored circles, and major metabolites are marked with a red border. Unfilled green circles indicate N/A, i.e. the substance was not detected, either because the substance was present in the sample at a level below the instrumental detection limit or because the substance was not present in the sample.

Based on the previous results and analyses, the main DEMs and candidate genes were simultaneously mapped onto KEGG pathway maps. This approach aimed to provide a clearer understanding of the relationship between metabolites and genes. For better illustration, we mapped the simplified carotenoid biosynthesis pathway (**Figure 6**).

**Figure 7:**
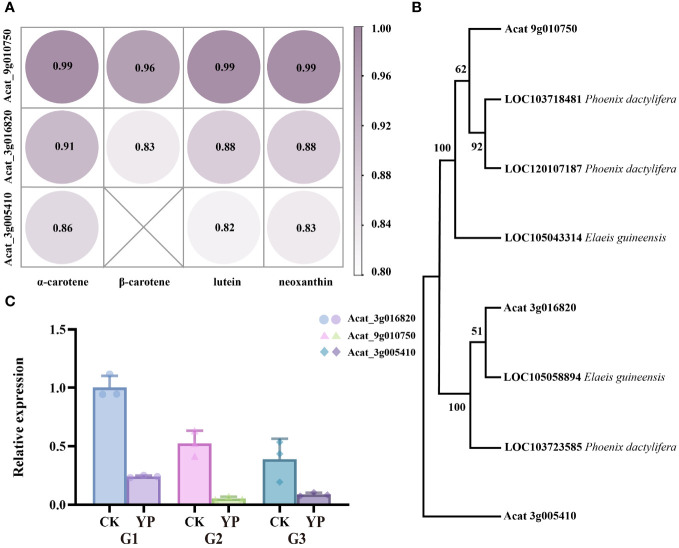
**(A)** Pearson correlation coefficients of candidate genes and major metabolite(α-carotene, β-carotene, lutein and neoxanthin). **(B)** Phylogenetic tree of *Areca catechu* L. candidate genes with *Elaeis guineensis*, *Phoenix dactylifera*. **(C)** The expression levels of candidate genes of carotenoid metabolites were detected by RT-qPCR. Error bars are standard deviations, only positive error bars are shown, and 3 replicates per column were averaged. CK denotes control and YP denotes bagging treatment. G1, G2 and G3 represent candidate genes Acat_3g016820, Acat_9g010750, Acat_3g005410, respectively.

The correlation between the candidate genes and the main four DEMs (α-carotene, β-carotene, lutein, neoxanthin) was consistently greater than 0.8, with Acat_9g010750 having the highest correlation coefficient, approaching 1 (**Figure 7A**). In addition, we used the NCBI (National Center for Biotechnology Information) database to obtain genes from the palm family (*Elaeis guineensis*, *Phoenix dactylifera*) that showed high homology to the amino acid sequences of the candidate genes. We then constructed a phylogenetic tree containing the above genes using MEGA 11 software (Mega Limited, Auckland, New Zealand). Neighbor-joining method employed to infer evolutionary history (Bakker, 2005), and the bootstrap test was conducted with 1000 replicates. Markedly, the three candidate genes belonged to three different taxa (**Figure 7B**).

### 3.5 RT-qPCR (Real-time quantitative polymerase chain reaction) verification

In our study, candidate genes were validated by qPCR and the results were consistent with phenotypic measurements (**Figure 7C**). This alignment suggests that the analysis of candidate genes was reliable.

## 4 Discussion

Among the chroma-related indicators, L* represents brightness, with higher values indicating increased brightness. A positive value of a* stands for red, while a negative value stands for green. Similarly, a positive value of b* stands for yellow, and a negative value stands for blue. The greater the absolute value of a* or b*, the darker the color presented (Wen et al., 2023). In this experiment, the changes in L*, a*, b* parameters before and after bagging were highly significant, resulting in increased brightness and a shift in color from dark green to yellow after bagging. Moreover, there was a prominent reduction of more than tenfold in carotenoid content following the bagging treatment. Referring to the impact of bagging on fruit skin carotenoids, as suggested by Wu. Tang et al. (Tang et al., 2020; Wu et al., 2020), it has been proposed that the effect may be due to the deterioration of light in the bag. This reduction in light transmission inhibits the chlorophyll synthesis in fruit skin, promotes the chlorophyll degradation, and enhances the manifestation of carotenoid color, thereby effectively improving the fruit surface color. In addition, in some other studies of fruit bagging, it has been observed that bagging treatment led to increase weight and L* value of single fruit after bagging (Li et al., 2017). The fruit surface brightness was higher, while the contents of chlorophyll and carotenoids in peel were lower (Wu et al., 2020). These findings align with the results of phenotypic measurements in this study.

Carotenoids, which serve as auxiliary pigments for chloroplast photosynthesis, protect chlorophyll in the plant body from bright light and serve as precursors for the synthesis of ABA (abscisic acid). The biosynthetic pathway of carotenoids is now well understood (Zhu, 2020). Carotenoids can be divided into two categories according to the different chemical structures. One category is hydrocarbons (containing only carbon and hydrogen, with a cyclic or non-cyclic structure and devoid of oxygen elements), with β-carotene being a typical representative. The other category is the xanthophylls (containing oxygen-containing functional groups such as hydroxyl, ketone, carboxyl, methoxy), also known as oxygen-containing carotenoids, with lutein being a typical representative (Zhu et al., 2017). It is generally accepted that light is a primary environmental factor influencing the regulation of carotenoid metabolism. Light can influence carotenoid metabolism during plant growth and development through photomorphogenesis, photosynthesis and photoprotection. Carotenoids are primarily synthesized and stored in plastids, with chromoplasts being the primary type of organelle for storing carotenoids (Fang, 2020). The results of current study demonstrated that, during the optimal harvesting period of areca nut fresh fruits (120 days after pollination), the untreated areca nut exocarp exhibited a darker green hue, abundant in chlorophyll, displaying higher photosynthetic efficiency and carotenoid content. However, the use of a double-layered inner black and outer yellow paper bag created an environment almost devoid of light for areca nut fruits. Consequently, the exocarp lacked chlorophyll, hindering photosynthesis directly through the fruit, and leading to a significant reduction in carotenoids content. Similarly, substantial changes in chlorophyll and carotenoid contents were observed when grapefruit, cucumber, and kiwi fruits were bagged (Liao et al., 2019; Shan et al., 2020).

We preliminarily screened 1784 DEGs and 21 carotenoid-like DEMs, which were analyzed by transcriptomics and metabonomics. DEGs were related to many metabolic pathways and cell activities, including carotenoid biosynthesis pathway which we focus on. Combined with the factors such as correlation coefficient, KEGG annotation and gene expression, the selection range of candidate genes is narrowed. We selected three of them as candidate genes for verification and the results are consistent with the pigment measurement results. We integrated transcriptomics and metabonomics to analyze the effects of bagging on carotenoid biosynthesis and regulation of A. catechu, and to interpret this physical measure that can artificially intervene to change the color of the exocarp from the molecular level.

At the molecular level, the accumulation of carotenoids is typically regulated transcriptionally by genes associated with the carotenoid biosynthesis pathway. This regulation is particularly influenced by specific genes that act as rate-limiting factors in carotenoid biosynthesis, including but not limited to *PSY*, *PDS*, *LCYB*, etc. (Liu et al., 2015; Ma et al., 2017). Dynamic balance between biosynthesis and metabolic catabolism determines carotenoid accumulation in tissues. This equilibrium is controlled by the interplay of biosynthetic enzymes in the upstream pathway, which regulate the flux of carotenoid production, and catabolic enzymes in the downstream pathway, responsible for metabolite turnover (Ampomah-Dwamena et al., 2022). The regulation of the carotenoid pathway varies, affecting carotenoid biosynthesis and dictating the accumulation in both photosynthetic and non-photosynthetic tissues (Sun and Li, 2020). The enzymatic pathway for carotenoid synthesis is located within the plastid and is metabolically linked to the methylerythritol 4 -phosphate (MEP) pathway. This connection leads to the production of C20 compound geranylgeranyl pyrophosphate (*GGPP*), which serves as a shared precursor for the biosynthesis of gibberellins, tocopherols, and chlorophylls (Okada, 2000).

The initial enzyme in carotenoid biosynthesis is phytoene synthase (*PSY1/CrtB*), and typically, an increase in its expression leads to a significant enhancement in carotenoid production (Fraser et al., 2002; Lindgren et al., 2003). In the yellowish fruit landrace Huang Song of tomato, the expression of *crtB* compensates for the deficiency in phytoene synthesis due to the decreased activity of *PSY1* (Lin et al., 2021). The insertion of synthase *crtB* into a white cassava plant resulted in the accumulation of β-carotene (Chavarriaga-Aguirre et al., 2017). Besides, *crtZ* plays a key role in α-carotene, β-carotene and subsequent production of zeaxanthin in a range of pathways. Silencing the Capsicum *crtZ* gene resulted in the observation of yellowing in Capsicum fruits (Tian et al., 2014). It has been shown that the expression level of *crtZ* affects the efficiency of 6-hydroxy-3-ketoend formation. A *crtZ* mutant with rhodoxanthin-synthesis activity has been found to enhance the production of 6-hydroxy-3-keto-e-end carotenoid (Furubayashi et al., 2022). The 8’-hydroxylase is a key enzyme in the *ABA* metabolic pathway, and it is encoded by the *CYP707A* gene family. Members of the peach *CYP707A* family cross-regulate the process of flower bud dormancy. In apple seedlings, dehydration tolerance is affected by an inhibitor of ABA 8′-hydroxylase *CYP707A* (Kondo et al., 2012; Wang et al., 2016; Yang et al., 2020).

In this study, the identified candidate genes might be involved in the regulation of *crtB*, *crtZ* and *CYP707A* related pathways. Bagging might led to changes in upstream and downstream expression of the carotenoid pathway as light intensity decreased. The inhibition of the conversion of the precursor substance *GGPP* to phytoene pathway might occurred due to the suppression of *crtB* expression, which affected the subsequent α-carotene and β-carotene content. Moreover, the subsequent pathway experienced a reduction in product content, particularly lutein, attributed to the repression of *crtZ*. Meanwhile, *CYP707A* might be involved in the conversion of *ABA*.

From the study, it is concluded that light can play an essential role in regulating the expression of fruit pigmentation genes (Llorente et al., 2017). Bagging, in turn, plays a shading role on fruit, potentially inhibiting the synthesis process of chlorophyll and carotenoid. The combined results of transcriptomics and metabonomics analysis indicate a decrease in the expression of key genes in the carotenoid biosynthesis pathway, accompanied by a reduction in the content of metabolites to varying degrees. The present study suggests that light intensity emerges as a key factor influencing the observed differences in color and carotenoid content in betel nut exocarp during fruit growth.

## 5 Conclusions

In the study, a decrease in the expression of key genes related to *crtB*, *crtZ* and *CYP707A* in the carotenoid biosynthesis pathway, accompanied by a reduction in the content of metabolites such as α-carotene, β-carotene, lutein and neoxanthin to varying degrees. Bagging plays a shading role on fruit, potentially inhibiting the synthesis process of chlorophyll and carotenoid indicated that light intensity may appear as a main factor influencing the noted shift from green to yellow and the ensuing reduction in carotenoid content after bagging. Currently, most of the research on fresh areca nut fruit focuses on the research of fruit growth, composition content and variety difference, while the research on the color of fresh areca fruit is relatively blank. This study used bagging as a physical measure to alter the color of betel nut exocarp through human intervention, and combined with transcriptomics and metabonomics for the first time focused on the effect of bagging on carotenoids in the exocarp at the molecular level. Preliminary exploration was conducted through the carotenoid metabolism pathway, may advance our knowledge of color transformations in areca nut exocarp, providing new ideas for the study of areca nut fresh fruit color and new methods for improving areca nut fruit quality. For farmers, the method of bagging has a certain degree of operability and has potential for improving the economic value of fresh areca fruit commodities. For researchers, bagging measures provide new ideas and methods for human intervention in fruit growth, and provide references for the improvement of fruit quality and theoretical research.

## Data availability statement

The datasets presented in this study can be found in online repositories. The names of the repository/repositories and accession number(s) can be found below: NCBI Accession number is PRJNA1063595.

## Author contributions

XZ: Data curation, Formal Analysis, Investigation, Methodology, Software, Visualization, Writing – original draft, Writing – review & editing. LH: Conceptualization, Funding acquisition, Methodology, Project administration, Supervision, Writing – review & editing. BF: Investigation, Validation, Writing – review & editing. CP: Investigation, Writing – review & editing. AI: Writing – review & editing. YZ: Writing – review & editing. HC: Writing – review & editing. JY: Project administration, Supervision, Writing – review & editing. YY: Conceptualization, Methodology, Project administration, Supervision, Writing – review & editing.
