# Supplementary material for: Integrated transcriptomics and metabolomics analyses of the effects of bagging treatment on carotenoid biosynthesis and regulation of Areca catechu L
Source: Front Plant Sci. 2024 Apr 2;15:1364945. doi: 10.3389/fpls.2024.1364945 (PMC11018958; doi:10.3389/fpls.2024.1364945)
Supplement: Supplementary file 1 [file DataSheet_1.pdf]

Supplementary Material

Integrated Transcriptomic and Metabolomic Analyses of the Effects of Bagging Treatment on Carotenoid Biosynthesis and Regulation of *Areca catechu* L.

Xin Zheng [1,2,†], Liyun Huang [1,†], Benyi Fan [1,2], Chunlin Peng [1], Amjad Iqbal[1,3], Yujie Zhang [1], Hongman Chen[4], Jianqiu Ye[1,*] and Yaodong Yang [1,*]

*** Correspondence:**

Jianqiu Ye

yejianqiu@catas.cn

Yaodong Yang

yyang@catas.cn

Supplementary

Supplementary Table 1

Reagent / Drug name	Grade/Content	Brand / Manufacturer	Place of Production
acetone	AR	Guangzhou Chemical Reagent	China
calcium carbonate	AR,≥99%	Xilong chemical industry	China
quartz sand	AR	Macklin	China
RNAprep Pure polysaccharide polyphenol plant total RNA extraction kit	——	TianGen (DP441)	China
PastKing One-step Synthesis of Premixed Reagents by Removing the First Chain of Genomic cDNA	——	,TianGen (KR118-02)	China

Table 1 Drugs and Reagents Information

Supplementary Table 2

Name	Brand / Manufacturer	Place of Production	Model	Remark
vernier caliper	i-Quip	China	——	accuracy 0.02mm
electronic balance	SHIMADZU	Japan	AUY220	precision 0.0001g
the light meter	SMART SENSOR	China	AS823	——
quartz cuvette	Nanjing jiancheng	China	722	1cm light diameter
centrifugal machine	HERMLE	Germany	Z 32 HK	——
spectrocolorimeter	Konica-Minolta	Japan	CM-700d1	——
ultraviolet spectrophotometer	SHIMADZU	Japan	UV-1600	——
real-time fluorescence quantitative PCR instrument	ABI	USA	QuantStudio™ 6	——
Micro UV Spectrophotometer	Thermo	USA	NanfoDrop 2000	——

Table 2 Instrumentation used in the test.

Supplementary Table 3

Primer name	Sequence (5' to 3')
Acat_9g010750-F	TTTTCCCGAAGGTGAAGCC
Acat_9g010750-R	TGCCGATGCCGCAAGTAA
Acat_3g016820-F	TACTCAGGGATGTAGGAGAAGATGC
Acat_3g016820-R	CAATGACTGGTTTCGGGACG
Acat_3g005410-F	AGTATGCCCCATTCCCTGTC
Acat_3g005410-R	GCCTCCATATCTACTTGGTGCTA
Acactin-F	CGTTGTGCTCAGTGGAGGATC
Acactin-R	TGCAAGGATGGAACCACCAATC

Table 3 Candidate gene RT-qPCR primers and internal reference genes.
